# Cognitive behavioral treatment for Sexsomnia (CBT-S). Case report and literature review

**DOI:** 10.1017/S1092852925100710

**Published:** 2025-11-17

**Authors:** Ulises Jiménez Correa, Horacio B. Alvarez García, Leon Rosenthal

**Affiliations:** 1 Faculty of Medicine, National Autonomous University of Mexico, Mexico City, Mexico; 2 Continuing Education and Innovation Division, National School of Higher Education, Leon Unit, National Autonomous University of Mexico, Mexico City, Mexico; 3 Sleep Telemedicine at dr.leonrosenthal.com, Houston, TX, USA

**Keywords:** CBT-I, CBT-S, inadequate sleep hygiene, insufficient sleep syndrome, sexsomnia

## Abstract

Sexsomnia is an arousal disorder in which abnormal sexual behaviors and experiences (such as masturbation, sexual intercourse, loud sexual vocalizations) are manifested during NREM sleep and are followed by amnesia upon awakening from these episodes. Both behavioral and pharmacological interventions are available for management of these manifestations, but no clinical trials have been performed to determine their effectiveness or guide clinical management. The aim of this review and case report is to describe the effectiveness of Cognitive Behavioral Therapy for sexsomnia (CBT-S) in a 27-year-old male whose chief complaint was sexual behavior 2 to 3 nights per week. The initial clinical assessment yielded diagnoses of sexsomnia, insufficient sleep syndrome, and snoring. The components of CBT-S used in this case included sleep extension, sleep hygiene, and relaxation therapy (diaphragmatic breathing with autogenic training) employing client-centered Motivational Interviewing. The patient reported no recurrence of sexsomnia events during the first month of therapy, but experienced 1 recurrence between the third and sixth months of follow-up, which was likely triggered by sleep deprivation and following alcohol consumption. No further recurrence of sexsomnia events was reported while maintaining adequate adherence to therapy. As is the case for insomnia, CBT represents a viable therapeutic option for the management of sexsomnia.

## Introduction

Arousal disorders are conceptualized as a dissociation between different brain regions characterized by an activation of the “central pattern generator” of basic behaviors (such as eating, sex, or aggression), accompanied by sleep inertia and sleep-stage instability.^1^ The International Classification of Sleep Disorders (Revised Text) describes sexsomnia as a specific subtype of arousal disorder in which sexual activity occurs during a partial arousal from NREM sleep and is associated with subsequent amnesia. Obstructive sleep apnea (OSA), periodic leg movements (PLMs), and sleep-related bruxism are recognized as potential triggers of sexsomnia episodes due to their association with sleep fragmentation and arousals.^2^

In a literature review, Schenck ^3^ reported a male predominance and consistent amnesia for sexsomnia episodes, with the main behaviors being sexual vocalizations, masturbation, fondling, and intercourse or attempted intercourse. He also identified OSA as a potential trigger for sexsomnia and noted that CPAP therapy can be an effective adjunct treatment in patients with comorbid sleep-disordered breathing, often reducing or resolving sexsomnia behaviors.

In addition to the possible association with other comorbid sleep disorders, it may be triggered or exacerbated by the consumption of illicit drugs ^4^ ^,^^5^ such as marijuana and medications used for other sleep disorders, notably sodium oxybate for narcolepsy.^6^ Marijuana and sodium oxybate have been reported to precipitate or worsen sexsomnia episodes, likely by disrupting sleep architecture and increasing arousals.

To date, a number of sexsomnia case reports have been published; for example, Peñas Martínez et al.^7^ published a case report of a 33-year-old male with a history of high blood pressure and medical antecedents of intrinsic asthma and nasal polyposis. The patient was reported to be a former smoker with no personal and familial antecedents of sleepwalking or other parasomnias. The patient’s main complaint was sexual sleep behavior, characterized by repeated attempts to remove clothing and touch his partner during sleep, without orgasm or ejaculation, persisting for 1 year. The neurological evaluation and PSG assessment only identified arousals lasting 3-4 seconds paired with diffuse EEG theta activity (4-5 hz). After 2 months of therapy with Clonazepam (1 mg), the patient reported decreased frequency of events despite intermittent adherence to treatment.

In another report, Yeh and Schenck^8^ published the case of a 20-year-old male soldier (with a history of childhood sleepwalking) with sleep masturbation and sleepwalking triggered by shift work and an irregular sleep–wake schedule due to military training. Clonazepam (0.5 mg at bedtime) was prescribed, which controlled sleepwalking but not recurrent masturbation. The patient decided to stop clonazepam, as he planned to leave military activities upon completing his obligation and expected to resume a regular and normal sleep–wake schedule. At the last available follow-up, the patient lived at home with his mother following a regular sleep–wake cycle; his mother reported no awareness of sleepwalking episodes, but no information was available about episodes of sleep masturbation, as he was sleeping alone.

Contreras, Richardson & Kotagal^9^ published a case report of a 16-year-old male patient with sexsomnia (masturbation during sleep), severe sleep apnea, and previous resection of a pinealoblastoma; following initiation of CPAP treatment, there was a substantial reduction in the frequency of sexsomnia episodes and daytime sleepiness. However, 6 months after the initiation of therapy, symptoms re-emerged despite continued CPAP treatment. Subsequently, a year of psychological therapy and hypnosis yielded a significant reduction in the frequency of sexsomnia episodes, as well as weight loss and amelioration of anxiety.

In another case report, a 42-year-old female patient experienced sleep-related sexual vocalizations (moaning, sexual phrases, and “dirty talk” not expressed while awake) that began during a period of significant workplace stress, with no evidence of sleep apnea or psychiatric disorders. Clonazepam 0.6 mg at bedtime reduced daytime sleepiness but did not affect episode frequency, leading to discontinuation of the medication. Interestingly, the episodes resolved completely after a job change to a lower stress position, despite ongoing sleep deprivation from family issues, underscoring the role of stress as a trigger and the importance of psycho-educational support for the patient and partner.^10^

The case of a 28-year-old male with sexsomnia and OSA had a clear clinical course.^11^ Symptoms of sexsomnia included masturbation, sexually fondling his wife, and inappropriately climbing on top of her during the night while sleeping. In this case, CPAP reduced sexsomnia episodes, but behaviors recurred after 3 weeks. While the addition of clonazepam and escitalopram was ineffective, CBT controlled the behaviors (though the authors did not provide the specifics of their CBT intervention). This case underscores that OSA-related arousals can trigger sexsomnia, CPAP often helps but may not fully suppress episodes, and CBT may be effective when medications fail.

The case of a 33-year-old male patient highlights the potential benefit of non-pharmacological interventions. He had a history of childhood parasomnias (sleepwalking and night terrors) and began having episodes of sexsomnia at age 18. Treatment with clonazepam was unsuccessful, and treatment with carbamazepine and lamotrigine (due to a clinical diagnosis of sleep hypermotor epilepsy [SHE]) was also discontinued due to ineffectiveness and increased frequency of events. The patient subsequently received stress management psychotherapy and hypnosis, which yielded a positive outcome, as the frequency of the episodes was decreased after 1 year of therapy.^12^

Kumar et al.^13^ also described the case of a 40-year-old man with chronic symptoms of sexsomnia, consisting of 2–6 events per month, ranging from kissing to sexual intercourse. He had experienced parasomnias since childhood, including sleepwalking and sleep talking, and reported that alcohol consumption worsened the episodes. On the PSG assessment, the apnea–hypopnea index was 1.3 events/h of sleep. No significant periodic limb movements during sleep, parasomnias, or abnormal EEG were observed. The authors reasoned in the description of the case that treatment with clonazepam would have similar effects to alcohol, which worsened the patient’s behaviors during sleep and thus elected to prescribe paroxetine. Complete resolution of symptoms for over 1 year was reported in this case.

There is strong evidence that psychological interventions improve sleep disorders, with CBT for Insomnia (CBT-I) having been shown to represent a viable treatment for both acute and chronic insomnia.^14^ For sleep disordered breathing (SDB), behavioral strategies such as weight loss, diet modification, fitness training, positional therapy (mostly avoidance of supine sleep), and alcohol avoidance (and at times other substances or medications such as benzodiazepines) reduce symptoms and daytime sleepiness.^15^ Also, non-pharmacologic therapies can reduce or replace medication in mild restless legs syndrome (RLS) or allow dose reduction in moderate or severe disease.^16^ In the case of sexsomnia, there is more limited documentation of the potential benefits of behavioral interventions, and robust evidence of their sustained effectiveness over time is lacking.

The present case report describes the effectiveness of Cognitive Behavioral intervention in managing sexsomnia over a 6-month follow-up in a patient presenting with comorbid insufficient sleep.

## Case report

The patient was a 27-year-old married male at the time of initial consultation, whose primary presenting symptom was sexual behavior during sleep (primarily masturbation and forced sexual intercourse) which disrupted sleep and led to significant marital difficulties. He reported a history of sleep talking and sleepwalking during childhood with no family history of parasomnias. Relevant aspects of the patient’s past medical history included asthma, tonsillectomy, and absence seizures (in remission, with normal EEG and no need for antiepileptic treatment for the previous 11 years).

During the first clinical consultation, the patient denied taking any medication and did not use any pharmacological treatment for sexsomnia during the course of CBT-S. He disclosed a history of sexsomnia episodes occurring 2 or 3 times per week, as reported to him by his wife. These episodes typically took place 2 to 3 hours after sleep onset, during which the patient remained asleep and was unresponsive to his wife’s attempts to interrupt the behavior or wake him. He reported no memory of these episodes, which were more frequent following sleep deprivation, alcohol consumption, and/or use of marijuana.

Additionally, dysfunctional sleep hygiene was identified due to delayed bedtimes because of extended work hours, computer and cell phone use. Consumption of caffeinated beverages in the late afternoon or evening, having late meals, occasional consumption of alcoholic beverages during evening parties, and smoking marijuana (3-4 times per year) were also reported.

Insufficient sleep syndrome (reduction of the scheduled time in bed resulting in a subjective sleep duration of 4.5 hours per night) and low risk of sleep apnea (as reflected on a STOP-BANG score of 3/8 due to snoring, tiredness/sleepiness, and male sex) were identified as comorbid sleep disorders.^2^ ^,^^17^

## Methods

The literature review was conducted in PUBMED, Scielo, Google Scholar, and in the BIDI UNAM database using the terms Non-REM parasomnia, Arousal Disorders, Sexsomnia, Therapy, and Treatment in English and Spanish. Articles describing treatments used in Sexsomnia cases were selected for inclusion.

Also, a case study was performed with pretreatment, post-treatment, and follow-up measures. Validated versions of the Epworth sleepiness scale,^18^ the Insomnia Severity Index,^19^ Patient Health Questionnaire 9,^20^ the Generalized Anxiety Disorder 7 scale,^21^ the WHO-Quality of life instrument,^22^ and a Spanish-translated version of the Paris Arousal Disorders Scale were applied.^23^ Sleep duration, subjective sleep quality, and efficiency were calculated based on structured clinical interviews.

The patient received psychological evaluation and a tailored CBT-S treatment protocol for sexsomnia (delivered by a Psychologist with expertise in Sleep Disorders) at a Sleep Disorders Clinic. Pharmacological intervention to address sexsomnia was not implemented, and the patient remained on no prescribed medications during therapy.

A tailored CBT-S protocol was implemented, consisting of Sleep Hygiene, Sleep extension,^24^ diaphragmatic breathing with autogenic training,^25^ and client-centered Motivational Interviewing (MI).^26^ ^,^^27^ MI was used to explore and address misconceptions and ambivalence regarding individual sleep requirements and dysfunctional beliefs related to various aspects of sleep hygiene practices. In this case, the focus was on stopping marijuana use, reducing alcohol consumption, increasing scheduled sleep times, and avoiding TV, computer, and cell phone use in bed.

In the first session, sleep education was provided covering sleep architecture, sleep homeostasis, and potential Parasomnia triggers. The legal risks associated with sexsomnia and the benefits of diaphragmatic breathing were also reviewed. Sleep hygiene measures emphasized maintaining a consistent and sufficient sleep schedule of 7.5 to 8 hours per night, discontinuing marijuana and alcohol use, regulating computer and cell phone use, optimizing the timing of daily meals, and allowing for brief daytime sleep (a 20-minute nap on weekends, if needed). The detailed CBT-S protocol can be seen in Table 1.

**Table 1.** Detailed CBT-S Protocol

Sleep extension		Increasing bedtimes to 7.5 to 8 hours per night (in a consistent sleep schedule, from 22:30 to 6:00 hrs on weekdays and from 23:30 to 7 hrs on weekends). If necessary, a 20-minute nap on weekends (before 16:00 hrs).
Sleep hygiene		
	Fitness	Incorporate fitness training at least 4 times a week for 20 t30 minutes (in the morning or afternoon) and preferably outdoors to facilitate exposure to natural light.
	Central Nervous System Depressants / Stimulants	Stop smoking marijuana and drinking soda, avoid alcoholic beverages after 17:00 hrs. Limit caffeinated foods and beverages to breakfast.
	Dinner	Light dinner without fat or spices, avoid eating within 2 hours of bedtime.
	Bedroom	Sleep in the dark (preventing outside light from entering the bedroom), use the bedroom only for sleep and sex (ie no television or cell phones), do not check the time in case of sleep difficulty.
	To facilitate sleep onset	Switch to the use of dim light 1 hour before bedtime and take a shower at 22:00 hrs.
Diaphragmatic breathing and autogenic techniques		The patient was trained in diaphragmatic breathing and autogenic techniques and asked to perform them just before going to bed (in the dark) and lying in bed during the early morning hours in case of insomnia.
Motivational Interview		Motivational interviewing (MI) is a strategy used to help the patient develop intrinsic motivation for behavioral change. It is based on a style of communication that blends patient-centered listening skills with methods directed at eliciting the patient’s motivation and commitment for change. MI happens in 4 overlapping processes that build on one another: engaging, focusing, evoking and planning.^27^

The first 3 sessions were held every 2 weeks. In the second and third sessions, subjective indicators of sleep quality, duration, and efficiency were reviewed. Scales and questionnaires were administered to assess clinical progress, and the patient’s questions were answered. Adjustments to sleep hygiene and sleep schedule practices were recommended, if warranted. As the patient reported no recurrence of sexsomnia during the initial month of treatment (between the first and third session), 2 online follow-up visits were conducted 3 and 6 months after the initial consultation.

Ethical considerations, including obtaining the patient’s informed consent and ensuring the privacy of the patient’s data, were addressed and properly documented in accordance with the standards of ethics and research committees of the Faculty of Medicine, National Autonomous University of Mexico.

## Results

The main symptom (sexual behavior during sleep) decreased from 2.5 episodes per week to a single event in the last 3 months of follow-up. In addition, pathological scores of insomnia, poor sleep quality, daytime sleepiness, depression, and low quality of life that were identified at initial consultation were all adequately responsive to CBT-S during the 6-month follow-up period (Table 2).

**Table 2.** Sleep Scales and Quality of Life

Scale	Pre	One month	Three months	Six months
*SexEv*	2*	0*	0**	1***
*ISI*	*11*	*5*	*4*	*3*
*PSQI*	*15*	*4*	*3*	*3*
*ESS*	*11*	*0*	*2*	*0*
*PADS*	19	8	0	7
*GAD–7*	*2*	*0*	*0*	*1*
*PHQ–9*	*10*	*1*	*0*	*0*
WHOQOL	*78*	*93*	*104*	*106*

Abbreviation: ESS: Epworth Sleepiness Scale; GAD-7: Generalized Anxiety Disorder-7; ISI: Insomnia Severity Index; PADS: Paris Arousal Disorders Scale; Pre: Pretreatment; PHQ-9: Patient Health Questionnaire-9; PSQI: Pittsburgh Sleep Quality Index; SexEv: Sexsomnia events (* per week, ** in the last 2 months, *** in the last 3 months); WHOQOL: World Health Organization Quality of Life Brief Version.

**Figure 1.**
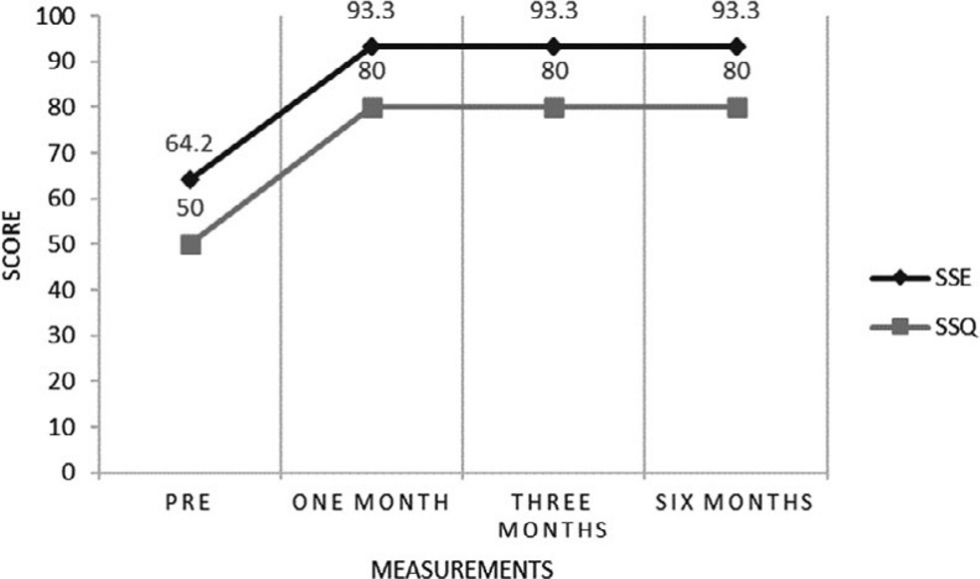
Subjective Sleep Efficiency and Quality. Pre, Pretreatment; SSE %, Subjective Sleep Efficiency %; SSQ, Subjective Sleep Quality (0-100). One Month: one month of follow-up. Three Months: three months of follow-up. Six Months: six months of follow-up.

Subjective sleep variables (Table 3) showed an important increase in reported sleep time, from 4 hours/night at initial consultation to an average of 7 hours/night, which was maintained during the last 5 months of follow-up. The number of awakenings per night also decreased, from 3 per night at baseline to zero per night at the 6-month follow-up visit. In addition, important improvements in subjective sleep efficiency and quality were documented once CBT-S was initiated (Figure 1).

**Table 3.** Subjective Sleep Quality

	Pre	One Month	Three Months	Six months
*SST*	*4.5*	*7*	*7*	*7*
*SSL*	*1*	*4.5*	*4.5*	*5*
*AW*	*3*	*0*	*1*	*0*

Abbreviation: AW, # of reported awakenings per night; SSL, Subjective Sleep Latency (minutes); SST, Subjective Sleep Time (hours).

## Discussion

An adequate response to CBT-S was documented in this case of Sexsomnia, with a decrease in the frequency of reported episodes from 2.5 episodes / week at initial consultation to a single episode reported during the last 3 months of follow-up. This constitutes an almost complete remission of the manifestations of sexsomnia experienced by the patient in response to CBT-S.

This is the first case report in which significant clinical improvement was achieved with CBT specifically tailored for sexsomnia, while acknowledging that Mioc et al.^12^ also reported favorable outcomes using a non-pharmacological intervention (psychotherapy and hypnosis) in their case report. This finding is particularly important, as long-term treatment with psychotropic drugs (mainly clonazepam) may, over time, contribute to confusional arousals and potentially become a priming factor for NREM parasomnias.^24^

Sleep deprivation is among the main priming and precipitating factors of arousal-related parasomnias,^5^ ^,^^24^ which justifies the emphasis on sleep hygiene and the importance of consistent bedtimes and sufficient sleep; hence, sleep extension has been used as a treatment option in the management of arousal parasomnias.^29^ In this regard, and most relevant to the present clinical report, characterizing the duration of nighttime sleep and identifying Insufficient Sleep Syndrome enabled the implementation of Sleep Extension and allowed for documentation of its benefits at follow-up.

In this regard, and based on this clinical report, it is important to characterize the patient’s sleep schedule practices and consider whether a diagnosis of Insufficient Sleep Syndrome is present, as Sleep Extension may represent a critical therapeutic intervention in addressing the patient’s presentation and is an important variable to monitor in the patient’s clinical progress.

Adequate sleep hygiene is one of the mainstays of non-pharmacological treatment of sleep disorders (and MI has been used as an adjunct to CBT and to improve adherence to sleep hygiene ^30^ ^,^^31^). However, this is overlooked or only briefly mentioned in the available literature. For example, rotating shift work is identified in 1 of 4 cases reported by Arino et al.^32^ In other reports, the importance of establishing a regular sleep schedule, reducing the consumption of energy drinks and alcohol, as well as controlling stress factors, is identified as beneficial and providing a decisive therapeutic effect.^33^ Therefore, dysfunctional or maladaptive sleep hygiene patterns in patients with Sexsomnia must be identified and targeted as one of the main components of psychological treatment. Identifying these features is critical to the success of a tailored sleep hygiene treatment plan.

The patient’s total anxiety score was low at the time of initial consultation, and anxiety was not identified as a relevant clinical concern. Nevertheless, in the context of the clinical diagnosis of Insufficient Sleep Syndrome, diaphragmatic breathing/autogenic training was encouraged to be performed (just before going to bed, in the dark, and when lying in bed during the early morning hours in case of insomnia) with the aim of contributing to sleep extension. This may have additionally contributed to controlling episodes of sexsomnia.

The pharmacological treatment of sexsomnia has been centered on the use of Clonazepam. While Schenck et al.^28^ ^,^^34^ initially reported high effectiveness of Clonazepam (9/10 cases), subsequent cases have reported only partial (or no improvement). The lack of adequate therapeutic response has, in some cases, prompted the discontinuation of the pharmacological intervention.^7^ ^,^^8^ ^,^^10^ Secondly, in the case of insomnia, it is important to acknowledge that benzodiazepines are ideally reserved for short-term management in order to minimize risks such as tolerance, withdrawal symptoms, and the potential for dependence associated with the prolonged use of these medications.^35^ ^,^^27^ Furthermore, chronic benzodiazepine use in insomnia is associated with shorter N3 and longer N1 sleep, along with altered EEG synchrony (reduced delta, increased sigma/beta, and enhanced spindling).^36^ These microarchitecture changes are more pronounced with higher doses and longer use and are linked to impaired arousal dynamics, increased nocturnal awakenings, and reduced sleep quality. It is thus not surprising that the current insomnia guidelines recommend avoiding, if possible, chronic benzodiazepine use ^14^ due to these sleep disruptions and associated risks.

Melatonin has been used with some success in the management of some parasomnias but has rarely been mentioned in the management of sexsomnia despite having a favorable safety and tolerability profile over Clonazepam and limited potential for drug interactions in a case report by Bras J. et al.,^37^ Melatonin 3 mg seemed to show a favorable therapeutic effect when used in conjunction with behavioral therapy. Also, an association between delayed sleep–wake phase disorder and arousal disorders has been suggested among Japanese students ^38^; and in a case report, with delayed sleep phase disorder, melatonin-controlled episodes of sleepwalking and sleep terrors (probably as a result of the correction of the circadian disorder and minimization of sleep deprivation ^39^). Therefore, the use of melatonin may represent a viable pharmacological option for some patients with sexsomnia and comorbid circadian rhythm disorders. This hypothesis deserves further study.

Clearly, in the case of pharmacological options for the treatment of sexsomnia, the lack of adequate options and placebo-controlled trials (in contrast to other sleep disorders) represents a disadvantage in the effective management of this condition. There is a need for the development of evidence-based treatment algorithms to help manage this specific type of parasomnia. These algorithms need to incorporate non-pharmacological treatment interventions, which also require further characterization. While limited data are available on non-pharmacological interventions, increasing evidence suggests the beneficial effects of psychological therapy and hypnosis,^9^ psychoeducational intervention,^10^ and CBT.^11^ Importantly, these interventions convey none of the risks and/or side effects associated with the chronic use of benzodiazepines.

The lack of polysomnographic assessment represents a limitation in the full portrayal of the present case report. However, the use of structured interviews, validated scales, and questionnaires enabled adequate diagnostic characterization and documentation of a favorable therapeutic response (which few case reports have previously documented over a period of several months). These tools enabled us to rule out the potential contribution of other comorbidities such as shift work disorder and sleep apnea ^8^ ^,^^11^ to the manifestations of sexsomnia.

A particularly relevant aspect of the polysomnographic assessment of these cases is the use of video-polysomnography, as it enables to rule out REM sleep behavior disorder (RBD), and/or nocturnal epilepsy. In this context, the patient only reported involuntary sexual behavior in the Paris Arousal Disorders Scale (without memory of these episodes nor related dreams, as it is experienced in RBD). Regarding a possible diagnosis of nocturnal epilepsy, the patient’s previous EEG recordings showed no evidence of electrical abnormalities; furthermore, in this case, sexsomnia episodes occurred within the first 2 hours of sleep (as has been described in arousal disorders) unlike nocturnal epilepsy which occurs at any time of the night. Finally, RBD or nocturnal epilepsy could have hardly been controlled without pharmacological intervention.

Polysomnographic assessment in the evaluation of sexsomnia represents an important consideration. Particularly among patients with a high index of suspicion of having other sleep-related comorbidities or for those who do not respond adequately to initial treatment. In the present case, snoring was not the patient’s chief complaint and the initial anamnesis clearly indicated the relevance of sleep deprivation and inadequate sleep schedule practices, which prompted prioritizing therapeutic intervention to address these maladaptive behaviors. While previous reports have identified OSAS as a possible causal factor of sexsomnia, in the present report, the patient’s total score on the STOP-BANG Scale (at the time of consultation) was 3 (only snoring, sleepiness, and male gender were positive). Importantly, excessive daytime sleepiness responded adequately to CBT-S as the scores on the Epworth Sleepiness Scale decreased following the initial assessment and remained negative (between 0 and 2 points) throughout the follow-up period. Even though a diagnosis of OSA was not formally ruled out with a PSG study, the patient’s clinical progress and resolution of symptoms did not warrant further PSG investigation. Nevertheless, long term follow-up is essential, as video-polysomnography may be required in the future. Particularly if CBT-S losses effectiveness, if the patient experiences recurrence of symptoms, if snoring worsens, or if other sleep-related symptoms emerge.

In summary, the patient’s clinical response to CBT-S was adequate and sustained throughout the available follow-up period; the outcome of CBT-S in this case demonstrates the benefits from psychological and behavioral interventions. However, we recognize the limitations of case study reports such as the limited generalizability and the need for robust clinical trials. The low prevalence of sexsomnia necessitates multicenter studies to validate findings and guide management.
